# Factors Influencing People's Willingness-to-buy Insecticide-treated Bednets in Arbaminch Zuria District, Southern Ethiopia

**DOI:** 10.3329/jhpn.v29i3.7867

**Published:** 2011-06

**Authors:** Frehywot Eshetu Gebresilassie, Damen Haile Mariam

**Affiliations:** ^1^CDC-Ethiopia, PO Box 11531, Addis Ababa, Ethiopia; ^2^Addis Ababa University, PO Box 9086, Addis Ababa, Ethiopia

**Keywords:** Community-based studies, Cross-sectional studies, Insecticide-treated bednets, User-fee, Malaria, Willingness-to-pay, Ethiopia

## Abstract

Promoting self-financing healthcare helps restore efficiency and equity to national health systems. This study was conducted in malaria-endemic areas of southern Ethiopia to assess the bednet possession of the community, determine the people's willingness-to-pay for insecticide-treated bednets (ITNs), and identify what factors influence it. The study provided relevant information for programme planners and policy-makers for evidence-based decision-making. This quantitative cross-sectional community-based study was conducted in four selected malarious Kebeles of Arbaminch Zuria district using a pretested interview-administered structured questionnaire. In total, 982 household heads were interviewed. The community's willingness-to-pay was assessed by contingent valuation technique using binary with follow-up method. The advantage, the distribution, and the payment mechanism were explained, and three different qualities of ITN were shown by constructing a hypothetical market scenario. Of the 982 respondents, 466 (47.5%) households had at least one functional bednet. Of 849 children aged less than five years in the 982 households, 185 (21.8%) slept under a net the night preceding the survey. The results of the study revealed that around 86% of the respondents were willing to buy ITNs. The average maximum willingness-to-pay for three different types of bednets was statistically different. The maximum amount the people were willing to pay was US$ 3.3 for a blue conical ITN, US$ 3.2 for a white conical one, and US$ 1.7 for a blue rectangular ITN. The community's willingness-to-pay was significantly affected by gender, educational status, perceived benefit of ITN, previous source of bednet, and characteristics of bednet. The results showed that a significant proportion of the community people were willing to pay for ITNs. Therefore, introducing a subsidized ITN market rather than free distribution for all should be considered to ensure sustainability and self-reliance in the prevention and control of malaria.

## INTRODUCTION

Ethiopia is one of the countries in sub-Saharan Africa seriously affected by malaria. About three-fourths of the total area of Ethiopia is malarious, and an estimated 48 million people—68% of the population—live in areas with risk of malaria (1).

Cognizant of this, the Federal Ministry of Health has developed a national five-year strategic plan for the prevention and control of malaria in Ethiopia (2). The target of the strategic plan was to attain a 100% coverage of all households in malarious areas, with at least one insecticide-treated bednet (ITN) by 2007 and that at least 90% of children aged less than five years (under-five children) and pregnant women will be sleeping under ITNs by 2010 (2). The strategic plan is to be implemented by providing subsidies to those not able to pay and by charging fees for the remaining population. This is because, like many other African countries, the Government is adopting user-fees and promoting self-financing healthcare to help restore efficiency and equity to national health systems. User-fees in the public-health facilities help promote equity because the demand for healthcare rises disproportionately with income. People who are well-off are more able and willing to pay for costly services. So, charging wealthier people for service they demand and can afford and pooling those revenues to subsidize those least able to afford care is a way to improve healthcare-delivery to the poor (3). ITNs are new to many people, and many are vulnerable to malaria but are too poor to afford buying ITNs. The Government also cannot afford to give free nets to all. Hence, a subsidized ITN market needs to be established since subsidized sales of ITNs can effectively introduce the component of sustainability and self-reliance in the prevention of malaria.

Willingness and ability-to-pay (WTP) refers both to preference and behaviour. The preference of consumers is formed based on three considerations: a consumer needs and desires; information about the existence and characteristics of a good or service and judgment about ones own probable benefit from that good or service relating to one's other needs and desires; and capacity to satisfy them, given the price and the cost of the transaction. Thus, when one speaks of studying the WTP for healthcare, one is talking about studying consumers’ demand for healthcare services (4). Therefore, assessing the people's WTP for ITNs and the factors influencing it would generate vital information that can make important contribution to the evidence-based design of malaria-control policies and strategies.

The objective of the study was to assess the bednet possession of the community in Arbaminch Zuria district, determine the people's willingness-to-pay for ITNs, and identify what factors influence it.

## MATERIALS AND METHODS

This cross-sectional community-based study was conducted in four randomly-selected malarious Kebeles of Arbaminch Zuria district of Gammo Gofa zone, southern Ethiopia. The study tool included a pretested interview-administered structured questionnaire. Two independent translators translated the questionnaire into the local language *Gammogna*, and the consistency was checked. The head of the household or one adult member representative of the study household was interviewed using the local language. Both data collectors and supervisors were able to speak the local language fluently. To assess the people's willingness-to-pay, contingent valuation using binary with follow-up method was used. The advantage, the distribution, and the payment mechanism were explained, and three different qualities of ITNs—one freely-distributed and two socially-marketed nets of different colours—were shown.

The two socially-marketed nets were conical in shape and, in terms of colour, one is white and the other one is blue. These nets were made available to the people with subsidized price by non-governmental organizations (NGOs) working in social marketing. The freely-distributed net is rectangular in type and blue in colour. It was freely distributed through support from the United Nations Children's Fund to all households living in malaria-endemic areas

The calculated total sample-size was 990 households using the formula:

n=Z^2^p (1-P)/d^2^

taking the price of an ITN as the main determinant for WTP,

where P=proportion of households who are willing to pay for an ITN at an average price of US$ 2.9 (estimated to be 25% referring to a survey by the Ethiopian Ministry of Health where WTP at US$ 2.6-6.2 is 28%), at 95% confidence interval (CI) and d=4%; it gives a sample-size of 450. Adding a design effect of 2 and non-response rate of 10%, the total sample-size was calculated to be 990.

Of the nine malaria-endemic Kebeles of Arbaminch Zuria district, four were selected by simple random sampling, and every 5th household was taken using systematic random sampling. Kebele (village) is the smallest administrative unit in Ethiopia, with an estimated 1,000 households living in it. The first household closest to the main road was taken as a starting point. The data-collectors with a minimum qualification of high school diploma were imparted a three-day training before their deployment. Two supervisors from the district health office checked the data-collection process daily.

The data were entered into the Epi Info software (version 6.04) and were cleaned and exported to the SPSS software (version 11.0) for analysis. Frequencies and measures of variation were used for describing the study population in relation to sociodemographic and other relevant variables. The strength of association and statistical significance between independent and dependent variables were assessed using crude odds ratio (with 95% confidence intervals (CI) and p value). Binary logistic regression analysis was performed using the SPSS software (version 11.0) to control for the potential confounding variables.

### Ethical considerations

Ethical approval was obtained from the Institutional Review Board at the Faculty of Medicine, Addis Ababa University. A support letter was obtained from the regional and zonal health administrations after a discussion on the significance of the study. Informed oral consent was obtained from every participant of the study after giving adequate information about the purpose of the study. Health education about different methods of prevention of malaria and about proper use of ITN was provided for all participants.

## RESULTS

### Sociodemographic variables

Of the calculated total sample-size of 990 households, 982 (99%) owners responded to the questionnaire. Of the household owners, 643 (65.5%) were male, and 339 (34.5%) were female. The mean age of the respondents was 40.8 years [standard deviation (SD) of 14.3 and range of 16-100 years]. Around 580 (63.9%) of the 908 respondents had a monthly family income of less than or equal to US$ 12.5; 319 (35%) earned a monthly income of US$ 12.5-62.5; and the remaining few (0.99%) earned a monthly income of greater than US$ 62.5 [The prevailing exchange rate used at the time of data-collection was US$ 1=ETB 8]. The monthly average total household expenditure was US$ 20.2, and the median total household expenditure was US$ 13.7.

### Possession of insecticide-treated bednets and related issues

Of the 982 respondents, 466 (47.5%) households had at least one functional bednet. Within the 466 households with at least one bednet, there were 556 nets. Thus, the mean number of nets per household in the study site was estimated to be 0.6 (n=982). The mean number of individuals per net for the 466 households (estimating 5 family members per household) would be 4.19 (n=556). Of the 849 under-five children in the 982 households, 185 (21.8%) slept under a net the night preceding the survey.

The common reasons mentioned for not having ITNs were unavailability of the bednets even for sale (36%, n=186/514), cannot afford to buy (30.4%, n=157/514), and lack of awareness about its use (10.9%, n=56/514). About 21% (n=514) of the respondents did not own ITN because they were waiting for a free distribution, and a few res-pondents (1.2% of 514) did not have one because they believed that using bednets does not prevent malaria

Of the 466 families who owned ITNs, around 248 (53.2%) received their ITNs free of charge, and 218 (46.8%) obtained these on payment basis from the local pharmacies, of which 198 (90.8%) paid US$ 2-3.1 for a single net.

### Willingness-to-pay characteristics of respondents

Of the respondents, 850 (86.5%) were willing to buy an ITN (if supplied by the market), and the remaining respondents were not (Table 1). Of those who were not willing to buy, the common reasons for their unwillingness-to-buy were inability to afford (40.9%, n=54/132), and 39 (29.5%, n=132) believed that they do not have to buy it since some people are getting ITNs free of charge. On the other hand, 17% (n=132) of the household owners were unwilling to buy as they did not have confidence in using bednets because they have observed that other people were getting sick of malaria while using bednets all the time. The remaining 17 (12.8%, n=132) res-pondents thought that they have enough bednets for the family and do not want any more.

**Table 1.** Willingness-to-pay characteristics of respondents in Arbaminch Zuria district, March 2006

WTP characterstics	Socially-marketed blue conical ITN	Socially-marketed white conical ITN	Freely distributed blue rectangular ITN
Willing to buy ITN, no. (%)	850 (86.5)	846 (86.2)	760 (77.4)
Average maximum WTP (US$)	3.3	3.2	1.7
Median maximum WTP (US$)	3.1	2.5	1.9

ITN=Insecticide-treated bednet;

WTP=Willingness-to-pay

**Fig. 1.**
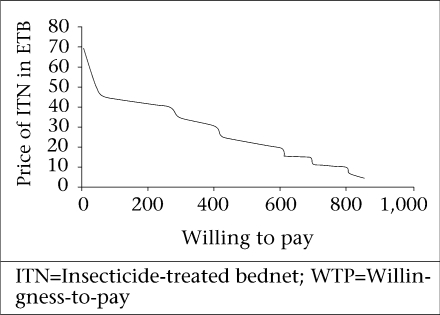
Demand curve for maximum WTP for a blue conical socially-marketed ITN in Arbaminch Zuria district, March 2006

**Fig. 2.**
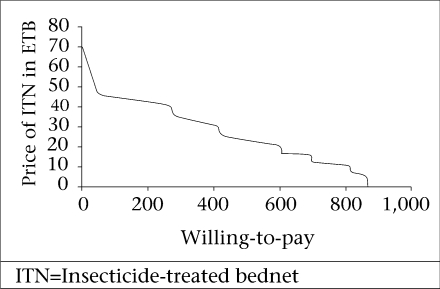
Demand curve for maximum willingness-to-pay for a white conical sociallymarketed ITN in Arbaminch Zuria district, March 2006

**Fig. 3.**
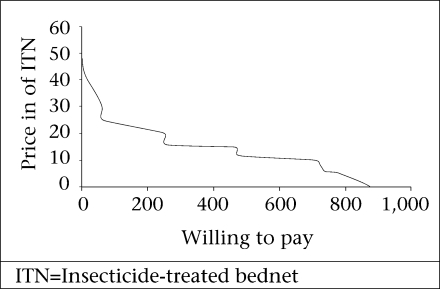
Demand curve for maximum willingness-to-pay for a blue rectangular freelydistributed ITN in Arbaminch Zuria district, March 2006

The maximum willingness-to-pay for three different qualities of ITN was evaluated. Fig. 1-3 show the demand curve for these ITNs using the cumulative WTP. The average maximum WTP for the blue conical ITN was US$ 3.3 (CI 3.17-3.39) and that of the white conical one was US$ 3.2 (CI 3.1-3.3), and this was found to be significantly different at the p value of 0.000. The average maximum WTP for the blue rectangular net was US$ 1.7.

A stepwise logistic regression was done to identify the factors which affect the individual's WTP. It showed that gender, educational status, farm-size holding, and perceived benefit of ITN were significantly associated with the individual's WTP. Table 2 shows that the females were 0.47 times less likely to be willing to pay for ITNs than males; this was statistically significant (p=0.03) even after controlling for the possible confounders. The educational status of the household head was also associated with their willingness-to-pay. People who could only read and write were almost three times (p=0.000) and those who finished elementary school were 3.3 times (p=0.014) more likely to be willing to pay than illiterate ones. The source of bednets of ITN owners was associated with the current willingness-to-pay. Those families that obtained their nets by purchasing were 3.4 times more likely to be willing to buy than those that got it free of charge (p=0.000). The association became 2.34 times even after controlling for the potential confounders (p=0.01). A significant association (p=0.001) was also found with the use of ITNs. Those who used their ITNs were about four times more likely to be willing to buy than those who did not (p=0.001).

**Table 2.** Factors affecting individual's willingness-to-pay for ITN using binary logistic regression, Arbaminch Zuria district, March 2006

Variable	No.	Crude OR (CI)	Adjusted OR (CI)
Gender of the respondent			
Female	339	0.47 (0.33-0.69)	0.43 (0.2-0.9)
Male	643	1	
Occupation			
Farmer	632	1	
Housewife	160	0.55 (0.35-0.88)	
Marital status			
Married	698	1	
Single	48	0.42 (0.26,0.66)	
Educational status			
Illiterate	439	1	
Can read and write	148	3.1 (1.6-5.9)	
Elementary school	201	3.3 (1.8-5.9)	3.48 (1.29-9.4)
Secondary school	185	2.29 (1.34-3.9)	
Family-size			
4-6	516	0.63(0.4-0.9)	
>6	295	1	
Farm-size holding (hectare)			
No farm land	201	1	
<1.25	551		2.3 (1.1-4.8)
≥1.25	230	5.62 (2.7-11.5)	5.3 (1.64-16.9)
Possession of radio			
Yes	721	2.8 (1.9-4.1)	
No	261	1	
Roof of house			
Corrugated sheet	707	1	
Thatched roof	270	0.47 (0.32-0.69)	
Knowledge on malaria			
Less knowledgeable	470	1	
Knowledgeable	510	1.76 (1.2-2.56)	
Benefit of ITN			
Perceived	944	3.79 (1.9,7.7)	2.01 (1.7-6.9)
Not perceived	38	1	
Source of ITN			
Freely given	248	1	
Bought	218	3.4 (1.8-6.4)	2.34 (1.1-5.2)
Number of ITNs in the house			
1-2	456	8.5 (2.8-26.2)	48.9 (10.8-221)
≥3	10	1	
Last-night use			
Yes	400	4.83 (2.65-8.84)	3.78 (1.78-8.0)
No	66	1	

The table shows only the significant responses;

CI=Confidence interval;

ITN=Insecticide-treated bednet;

OR=Odds ratio

The common reasons mentioned for unwillingness to buy in the present study were also unaffordability and expectation of free distribution for all. Results of logistic regression showed that the monthly income of the households was not a significant determinant of people's willingness-to-pay.

**Fig. 4.**
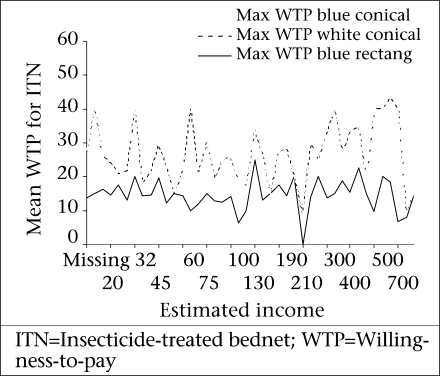
Mean willingness-to-pay for ITN versus estimated monthly income (ETB) (income elasticity of WTP), Arbaminch Zuria district, March 2006

Figure 4 shows the mean willingness-to-pay for the three different qualities of ITN versus estimated monthly income; the maximum WTP does not show a striking increment with a change in income.

## DISCUSSION

The coverage of bednets was 50% in a survey in Jimma area in southwest Ethiopia (5) and 80% in rural malarious areas of Kefta-Humera district in northern Ethiopia (6) whereas it was 48% in the present study. These figures are more than the one reported in the Ethiopian Demographic and Health Survey (EDHS) 2005 which was 36%, for any kind of bednet, among households in areas below 1,000 metres above the sea level (7). This may be due to the intensive free distribution being made in these sites after the EDHS 2005 was conducted.

The results of the present study revealed that around 87% of the respondents were willing to buy ITNs, if supplied by the market with a price proportional to their income. Similarly, in a study in western Shoa (central Ethiopia), 99% did not have prior experience of using any bednet but 96% of them were willing to pay for ITNs (8). A survey by the Ethiopian Ministry of Health showed that 40% of the population was willing to buy ITNs, and affordability was one of the determinant factors that impede the possession and use of ITNs. This becomes more severe in areas where the community lacks prior knowledge and culture of using bednets (9).

When one speaks of studying the WTP for healthcare, one is talking about studying consumers’ demand for healthcare services (4). The present study did not show the effect of income on WTP, although this should be interpreted cautiously as most people in Ethiopia are not open when it comes to disclosing their income levels. A review of contingent valuation studies in Europe has shown that income tends to influence willingness-to-pay positively and significantly. The elasticity estimates are, in most cases, greater than zero but less than unity (10). This was explained by the reason that poor people tend to spend as equal amount as those with better economic status to improve their health status since health improvement measures tend to be relatively more beneficial to low-income groups. This may also be true in our situation where malaria is mainly affecting the poor. Another study in Tanzania looked at the role of gender on WTP for ITNs and found that females were less likely to be willing to pay for ITNs with higher prices compared to males (11). In the present study also, gender was found to be one of the determinant factors for purchasing ITNs. Females were less likely to be willing to pay for ITN than males. Other predictors of willingness-to-pay for ITNs were educational status, farm-size holding, and the source of ITN. This partly goes in harmony with a study in Uganda where predictors were ownership of a television, being a skilled worker, and professional or owning major business and having better health-related belief (12). In a study in Jimma (southwestern Ethiopia), the independent predictors of purchasing had been possession of a radio, being knowledgeable, and occupation of the household head (3).

In the present study, among those families which already had at least one ITN, its source was also a significant predictor as people who obtained their ITNs by purchasing were twice more likely to buy another bednet than those who got it free of charge. In addition, those respondents who perceived the benefit of ITNs were also more likely to be willing to buy it, showing that favourable health beliefs were important not only in predicting the use of mosquito-nets but also people's willingness to buy it.

Apart from the social and economic background of individuals, the characteristics of ITN had a great impact on WTP as willingness varies with ITNs of different attributes (13). This was also observed in the present study. In this study, people showed more preference and were more willing to buy the conical type of socially-marketed ITNs than freely distributed rectangular ones. The reason for such a preference mentioned by most of them was that the rectangular ITN has ‘low quality’ than the conical ones. The common-sense notion is that quality, as perceived by the consumer, is a major determinant of demand for medical care services. The results of the present study also revealed that free distribution of ITNs had a negative influence on people's willingness to buy ITNs which subsequently may create a challenge on malaria-prevention efforts. Moreover, user-fee will stimulate the increasing availability of attractive branded qualities of ITN, its demand, and also use. After all, it is the perception of high quality that underlies a consumer'sperception of value of goods (4).

### Limitations

The present study has certain limitations. The test-retest method should have been used for increasing the validity of the contingent valuation and the fact that there was no complementary qualitative data-collection. However, due to constraints of time and other resources, these could not be done.

### Recommendations

The study has come up with important results to make the following recommendations:

- Proper information, education, and communication (IEC) messages should be designed on malaria to introduce proper health-related belief and demand for ITNs in the community and to prioritize the vulnerable group of the population.
- Specific interventions should target under-five children and pregnant women to incr-ease the coverage of bednets per household and to those vulnerable groups who are sleeping under bednet.
- Introducing a subsidized ITN market rather than free distribution for all (as there is willingness-to-pay for ITNs by a significant proportion of the population) to ensure sustainability and self-reliance in the prevention of malaria.
- The study revealed that people's willingness-to-pay for bednets was significantly affected by the perceived quality in terms of its shape and colour. Therefore, provision of bednet to communities should be made based on their preferences and taking into consideration their housing conditions.

## ACKNOWLEDGEMENTS

The authors acknowledge the Ethiopian Public Health Association for financially supporting this project and the Population Service International Ethiopia for supplying the socially-marketed ITNs for demonstration during data-collection. The authors extend their gratitude to the Arbaminch Zuria district health office for facilitating the field work and the community of Arbaminch Zuria district for participation in the study.
